# The direct anterior approach: initial experience of a minimally invasive technique for total hip arthroplasty

**DOI:** 10.1186/1749-799X-7-17

**Published:** 2012-04-25

**Authors:** Ola Hallert, Yan Li, Harald Brismar, Urban Lindgren

**Affiliations:** 1 Department for Clinical Science, Intervention and Technology (CLINTEC), Division of Orthopedics, Karolinska Institutet, Stockholm, Sweden

**Keywords:** MIS arthroplasty, Anterior incision, Complication, THR

## Abstract

**Background:**

Less invasive approaches for hip arthroplasty have been developed in order to decrease traumatisation of soft tissue and shorten hospital stay. However, the benefits with a new technique can be at the expense of a new panorama of problems. This manuscript describes, with emphasis on postoperative complications, our experience from the first 200 cases of unilateral hip replacement using the direct anterior minimally invasive (MIS) approach.

**Methods:**

A straight incision in front of the greater trochanter was used and the tensor muscle was approached subfascially and retracted laterally. The joint was opened and the femoral head was removed. Usually excellent acetabular exposure was obtained. In order to get access to the proximal femur, the hip capsule was released posterolaterally so that the femur could be lifted using a special retractor behind the tip of the trochanter. After insertion of the prostheses, the wound was closed using running sutures in the fascia overlying the tensor, sub- and intracutaneously.

**Results:**

There was a small influence of BMI on the duration of surgery, and obese patients tended to have the cup positioned at a higher degree of deviation. There were in total 17 complications of which 5 necessitated revision surgery; 3 peroperative femoral fractures and 2 dislocations. Another 4 dislocations were treated with closed reduction and did not recur. 3 cases of nerve injury were noted, all resolved within 12 months. Three cases of DVT were diagnosed as well as 2 cases of postoperative infection; none of these led to chronic disability.

**Conclusions:**

The technique is perhaps more technically demanding than the lateral approaches used today due to the somewhat limited surgical exposure. Morbidly obese or very muscular patients as well as patients with a short femoral neck or acetabular protrusion can represent particular problems. Our results indicate that there are certain risks when adopting this procedure but the complications noted are avoidable.

## Background

There is a strong tendency for surgical techniques to be improved over time and as new instruments are developed less invasive approaches are possible [1]. Currently the most often used surgical exposures for hip replacement such as the anterolateral and the posterolateral approaches involve splitting muscles with risk of partial denervation and detachment of tendons with a risk for incomplete healing. In many cases this results in weakness of hip abductor muscles and notable limp. Using the direct anterior approach for total hip arthroplasty these problems are largely avoided and several reports in the literature have documented advantages with the technique [2-6]. A short rehabilitation time due to minimal soft tissue trauma is often emphasized. Complications have also been encountered, such as injury to the lateral femoral cutaneous nerve, component malposition, damage to the femoral shaft and delayed wound healing [2-5,7]. Obviously, the benefits with the new technique are at the expense of a new panorama of complications and intraoperative difficulties. Exceptionally skilled surgeons might be the first to describe the results and when the technique is more widely adopted more problems can become apparent. This report describes the initial experience by a team of four surgeons, a senior surgeon, a recently board certified orthopaedic surgeon and two orthopaedic residents. The aim of the study was to describe early complications so that surgeons who adopt the technique will be informed not only about potential benefits but also of potential risks with the technique.

## Methods

### Surgical technique and clinical follow-up

**Figure 1:**
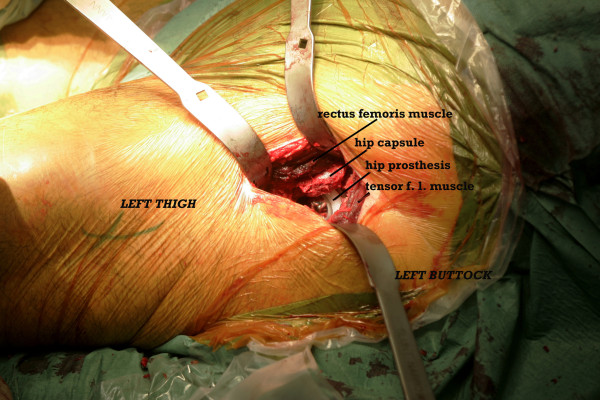
Intraoperative photograph.

This is a retrospective analysis of patient records and radiographs from the first 200 consecutive cases of unilateral hip replacement using the direct anterior approach in our department; the period at study was 1 year after surgery. All were primary, unilateral hip replacements and there were no exclusions with reference to age, obesity or diagnosis except that no case of severe deformity such as high hip dislocation was included. All operations were done using the same technique, a standard operating table and the patient supine. The hip was positioned at the table break in order to allow extension during the procedure. Both lower limbs were prepped and separately draped. A straight incision was placed approximately two finger breadths in front of the greater trochanter and extended proximally to the level of the anterior superior iliac spine. The subcutaneous tissue and the fascia over the tensor fascia lata was split the length of the incision and the medial margin of the tensor muscle was approached subfascially so that special Hohmann retractors could be placed deep to the muscle, one on the hip capsule and one on the vastus lateralis ridge of the greater trochanter. The joint was opened longitudinally, a segment of the femoral neck was removed and the femoral head extracted using a corkscrew. Usually excellent acetabular exposure was obtained using the special pointed retractors anteriorly and posterolaterally. After reaming, a hemispherical Ti alloy shell (Trident PSL™ with Ti porous coating and hydroxyapatite; Stryker USA) was fixed aiming at 10–20° of anteversion and 40–50° of deviation. In order to get access to the proximal femur, the hip capsule was released posterolaterally so that the femur could be lifted using a special retractor behind the tip of the trochanter. Broaching was facilitated by putting the leg into extension, adduction and external rotation. This was greatly facilitated by having an experienced assistant on the same side; to some extent this is also true for the second assistant on the opposite side. We used uncemented Ti alloy femoral components (Accolade™ with Ti proximal porous coating and hydroxyapatite; Stryker USA) and a 28 mm cobalt-chromium head. The instruments used were Stryker’s instruments for minimally invasive surgery of the hip (Figure 1). The wound was closed using running resorbable sutures in the fascia overlying the tensor muscle, subcutaneously and intracutaneously. The patients were allowed immediate partial weightbearing as tolerated using two forearm crutches as needed for 6–8 weeks. Clinical follow-up was carried out at 2 and 12 months post surgery, the latter also included x-rays.The study was approved by the Ethics Committee of Karolinska Institutet and was conducted in accordance with the Declaration of Helsinki.

The deviation of the acetabular cup was measured on pelvic AP-radiographs being the angle between a line joining the ischial tuberosities and the line through the ellipse described by the acetabular cup. The anteversion was calculated, using the method described by Ackland, Bourne and Uhthoff [8]. The findings could be compared to the mandatory postoperative X-rays.

### Statistical methods and data management

Multiple comparisons of continuous data were performed by analysis of variance, ANOVA. In the case of a statistically significant result in the ANOVA, statistical comparisons were made by use of the post-hoc test proposed by Fisher to control for multiplicity. The Pearson correlation coefficient was used in order to test independence between variables. In addition to that, descriptive statistics was used to characterize the data. In the case of a statistically significant result the probability value (p-value) has been given.

## Results

**Figure 2:**
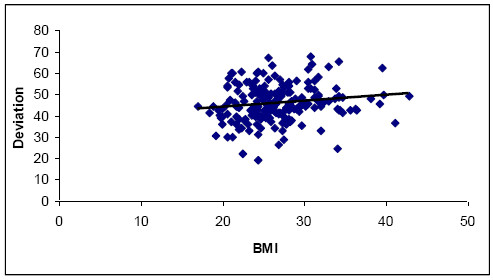
Correlation between BMI and deviation of acetabular cup.

**Figure 3:**
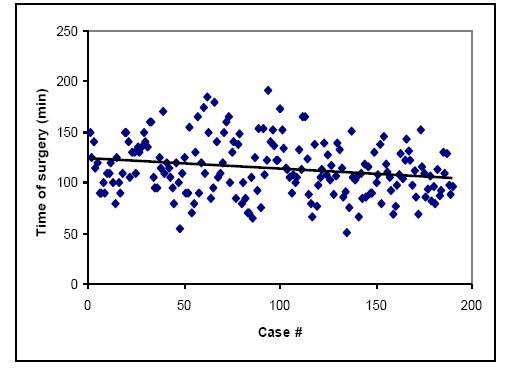
Peroperative blood loss.

The material is described in Table 1. Mean values for blood loss, operating time and cup position are shown in Table 2. There was a small but statistically significant influence of BMI on the time of surgery (Table 3). Similarly, obese patients tended to have the cup positioned at a higher degree of deviation (Figure 2). The estimated intra operative blood loss was not significantly correlated to the BMI. It did not change significantly in the whole material during the course of the study. The time of surgery decreased significantly with the experience of the team (Figure 3).

**Table 1:** Patient summary

**Patients (n = 200)**		
**Women**	121	
**Men**	79	
**Age**	29–88 (mean 67,4)	
**BMI**	17–43 (mean 26,7)	
**Diagnoses**	Osteoarthritis	152 (76%)
	Inflammatory arthritis	31 (15,5%)
	Avascular necrosis	9 (4,5%)
	Dysplastic osteoarthritis	8 (4%)

**Table 2:** Blood loss, operating time and cup position

	**Mean**	**SD**
Time of surgery (min)	114	28.9
Blood loss (ml)	496	322
Anteversion	18°	7,6°
Deviation	46°	8,5°

**Table 3:** Influence of the BMI

	**Increasing BMI**
Blood loss	n.s.
Operating time	y = 1,1179x + 85,113 R^2^ = 0,0317 P < 0,05
Anteversion	n.s.
Deviation	y = 0,2816x + 38,473 R^2^ = 0,0238 P < 0,05

**Figure 4:**
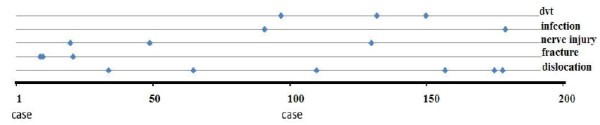
Postoperative complications.

Femur fractures in the early postoperative period occurred in cases 9, 10 and 21 (Figure 4). The cause of the cracks in the proximal femur were likely due to improper broaching. These patients were revised using a standard anterolateral approach; a wire cerclage was used to fix the fractures. In one case the same prosthesis was used, but in case number 10 a long-stem (Wagner™, Zimmer USA) prosthesis was used together with a cemented cup and in case 21, a Meridian™ prosthesis (a cobalt-chrome prosthesis with proximal coating, Stryker USA) was used instead of the Accolade due to its better filling of the metaphysis.

There were six dislocations in the postoperative period, number 34 and number 67 were due to instability of the femoral component resulting in rotation and/or subsidence. These two cases underwent stem revision and in case 34 the cup was revised as well. The other four cases, two men and two women, were treated with closed reduction under anesthesia and dislocation did not recur. One of these cases had acetabular dysplasia and is shown in Figure 4. One superficial and one deep infection occurred (the latter was case number 193 caused by staphylococcus aureus; this patient underwent lavage shortly after the hip replacement. The infections healed after proper antibiotic therapy for 3 months (Table 4).

**Table 4:** Reoperations

**Complication (% of all)**	**Case no.**	**Treatment**	**Outcome**
Periprostethic femoral fractures (1,5%)	9 10 21	ORIF with wiring Total revision Stem revision	All healed
Dislocations (1%)	34 67	Total revision Stem revision	No further dislocation
Deep infection (S.Aureus) (0,5%)	193	Irrigation and antibiotic therapy	Resolved, prosthesis retained

Three cases of DVT were diagnosed (case number 103, 140 and 160), all in women; number 103 also had a pulmonary embolus. There were three cases of nerve injury. Number 20 had dysaesthesia on the lateral thigh, likely due to peroperative damage to the lateral femoral cutaneous nerve. Number 49 was noted to have decreased peroneal nerve function and number 138 had an almost complete loss of femoral nerve function postoperatively. All three cases had resolved at 6 months postoperatively (Table 5).

**Table 5:** Closed treatment/observation only

**Complication (% of all)**	**Case no**	**Treatment**	**Outcome**
Injury to lat. fem. cut. n. (0,5%)	20	Observation	Resolved
Peroneal n. palsy (0,5%)	49	Observation	Resolved
Injury to femoral n. (0,5%)	138	Orthosis	Resolved
Dislocation (2%)	118; 169; 189; 192	Closed reduction	Stable

There was no significant correlation between complication and gender, age or BMI.

## Discussion

The introduction of new approaches and instruments can be expected to temporarily be associated with complications, such as neurovascular injury and component malposition. It is important to have information about the risk for various complications and predisposing factors. This study is based on non-selected, consecutive, primary unilateral total hip replacements. The very reason to use this approach is the possibility to minimize surgical soft tissue injury and to maintain normal muscle function and stability of the hip [1,7,9-13]. The approach is internervous and does not include the release of muscles or tendons. In the literature there is little evidence for a higher complication rate with the direct anterior approach compared to lateral approaches. It is however of interest to demonstrate and discuss the hardships involved when the technique is adopted by a team of surgeons with mixed experience.

Overall the distribution of complications was fairly even among the 200 cases included in the study. Aside from the first 10 cases, this study does not well delineate a learning curve. An almost linear reduction in intraoperative blood loss was noted among the first 10 cases; in two of them cracks occurred in the proximal femur – this was not seen in cases done later.

**Figure 5:**
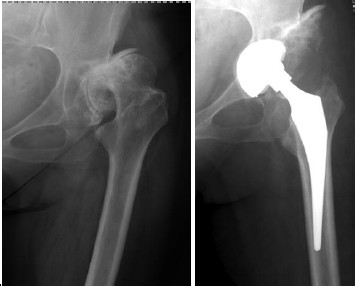
Radiograph of dysplastic hip that dislocated once.

Access to the femoral canal can be expected to be difficult especially in patients with a short and varus angulated femoral neck and where the range of motion is restricted due to fibrosis of the joint capsule. Other authors [3,12,14,15] have reported fractures in the proximal femur with this approach. In our series the major predisposing factor seems to have been pronounced osteoporosis. With the currently used offset handle, broaching is safer than with a standard straight handle as in the present series. The risk for fracture of the proximal femur is further minimized by adequate posterolateral capsule release so that an elevator can be placed around the tip of the trochanter while the hip is extended, adducted and externally rotated. However, if the surgeon excessively releases soft tissues from the proximal femur (which is not necessary) instability could result. This was possibly the reason for the dislocations that were seen in cases 118, 189 and 192 in our series. Even if no instability occurs, the release of tendons from the proximal femur counteracts the objective of this approach. Case number 169 was a patient with dysplasia (Figure 5) where the necessary medialization of the femur and not excessive soft tissue release led to postoperative instability. The end result of that operation was excellent.

The direct anterior approach subjects the lateral femoral cutaneous nerve and the femoral nerve to higher risk than lateral approaches. Even though the posterior branch of the lateral femoral cutaneous nerve can be injured [2,12,16] the technique described here seems to be effective in avoiding clinically relevant damage to the nerve. This is important since dysesthesia due to injury of this nerve may lead to considerable discomfort in the thigh for several months. Injury to the femoral nerve with the anterior approach has been mentioned by others [3,17,18]. We believe that it can be caused by the retractor placed anteriorly so that the iliopsoas muscle and the femoral nerve are compressed against fascial structures. This retraction should be directed cranially, towards the opposite shoulder and not transversely. In addition we have also constructed a retractor which sits around the ilium at the level of the anterior inferior iliac spine. This instrument is useful in difficult cases such as patients with pronounced abdominal obesity. Fortunately, femoral nerve injury seems to have a good prognosis and should resolve completely within a few months.

We decided to use the direct anterior approach even in cases with morbid obesity and in cases with grade 1 and 2 acetabular dysplasia. In obese patients we found that there was a specific problem in positioning the acetabular component. A high BMI was also associated with a longer operative time but not with a significantly higher intraoperative blood loss.

## Conclusions

In summary our intention with this report is to discuss the risks involved with the adoption of a MIS technique for total hip replacement. It has the theoretical advantage of minimal soft tissue injury and it has been shown that the expectations of facilitated early rehabilitation can be fulfilled [9,10]. In our opinion the present results do not obviate the technique as a routine for total hip arthroplasty but it is necessary to have a thorough understanding of the risks beforehand. Although technical difficulties necessitating revision surgery were more obvious among the early cases other complications such as dislocations may appear later. The technique is perhaps more technically demanding than the lateral approaches used today due to the somewhat limited surgical exposure. It should be reserved for specially trained surgeons who have the possibility to treat many patients in order to maintain good skills. It has been our overall impression that morbidly obese or very muscular patients as well as patients with a short femoral neck or acetabular protrusion can represent particular problems.

## Competing interests

Funds were received from Stryker® Sweden.

## Authors’ contributions

UL is the senior orthopedic surgeon. HB is the orthopedic specialist. OH and YL are residents. UL drafted the manuscript. OH and HB analyzed the data. HB and YL contributed to the manuscript preparation. All authors read and approved the final manuscript.
